# Lunar and Martian Regolith Simulants Desorb and Weather after Exposure to Bioregenerative Life Support System Effluent

**DOI:** 10.1021/acsearthspacechem.5c00267

**Published:** 2026-01-07

**Authors:** Harrison R. Coker, Daniella Saetta, Misle M. Tessema, Jackson L. Smith, Charles A. Richardson-Gongora, Jason A. Fischer, Hannah I. Roberts, Luke B. Roberson, Julie A. Howe

**Affiliations:** † Department of Soil and Crop Sciences, 14736 Texas A&M University and Texas A&M AgriLife, College Station, Texas 77843, United States; ‡ 564118 Bennett Aerospace, Kennedy Space Center, Merritt Island, Florida 32899, United States; § National Aeronautics and Space Administration, Kennedy Space Center, Merritt Island, Florida 32899, United States; ∥ Noetic Strategies, Kennedy Space Center, Merritt Island, Florida 32899, United States; ⊥ Aetos Systems, Kennedy Space Center, Merritt Island, Florida 32899, United States; # Genetics Institute, 3463 University of Florida, Gainesville, Florida 32610, United States

**Keywords:** ISRU, BLiSS, regolith, weathering, simulants

## Abstract

The extraction of plant essential nutrients from extraterrestrial regolith will be necessary to ensure the sustainability of lunar and martian agriculture. An essential instrument of these outposts will be bioregenerative life support systems (BLiSS) that attempt to fully recycle nutrients from organic wastes. While BLiSS may not be fully efficient and lead to a reduction in the quantity of some elements, it is necessary to explore if regolith can be used to fortify the composition of BLiSS effluent. Lunar (JSC-1A) and martian simulants (MGS-1) were reacted with a high-fidelity BLiSS effluent from NASA’s Kennedy Space Center (KSC) in a 24 h batch experiment and compared to reactions with an inorganic nutrient solution and water. Net sorption and dissolution of elements were determined by quantification of reacting solutions using inductively coupled plasma-optical emission spectroscopy (ICP-OES), with P demonstrating Langmuir and Zn and K demonstrating Freundlich sorption isotherms. The lunar simulant desorbed sizeable quantities of S, followed by Ca and Mg, while the martian simulant desorbed S, followed by Mg, Ca, and Na. Elemental bonding of C, N, P, and Ca was observed on the simulant solid phase with X-ray photoelectron spectroscopy (XPS) after reaction with BLiSS solution. Minerals after experimentation were observed using scanning electron microscope−electron dispersive spectroscopy (SEM-EDS), revealing pitting in JSC-1A and covering of nanoparticles in MGS-1. There were marked differences between the reactions of the inorganic nutrient solution compared to BLiSS effluent, indicating the necessity to study high-fidelity solutions over single-element model systems. Overall, lunar and martian regoliths contain highly soluble components that may fortify BLiSS effluents with valuable metals and plant essential nutrients.

## Graphical abstract

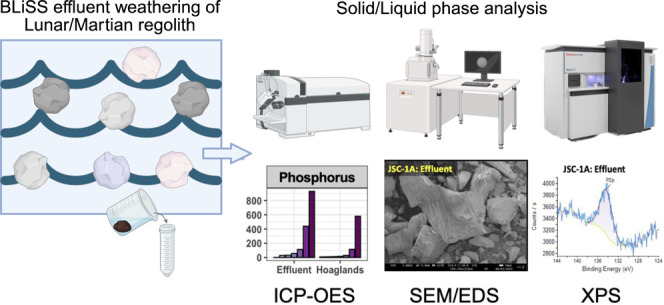

## Introduction

### Background

Inorganic nutrient solutions (e.g., Hoagland’s^1^) are useful for growing plants in soilless systems but require continuous nutrient inputs that are consumed as plants grow. With future plans for lunar and Mars settlements, dependency on Earth-sourced fertilizers will hinder efficient operations. Therefore, extracting nutrients from regolith to enhance sustainable agricultural operations, or improving the regolith’s fertility such that plants directly derive nutrients from the regolith, are both effective strategies that support diverse cropping operations to produce food and fiber resources through in situ resource utilization (ISRU).^2,3^ Broadly, approaches that utilize surface resources from the Moon and Mars to reduce inputs from Earth are deemed ISRU.^4^

### In Situ Resource Utilization

The extraction of raw elements from regolith is a primary goal of ISRU.^4^ Various approaches for enhanced dissolution of regolith have been studied,^5^ including techniques such as ionic liquids,^6^ eutectic salts,^7^ electro-deoxidation,^8^ and heat treatment.^9^ While promising, these approaches have the downfall of requiring exogenous chemicals, energy, and technology. Alternatively, a basic approach will encompass using resources such as BLiSS effluent to physically weather the regolith. Because of the underlying basaltic mineralogy of lunar and martian regoliths, hydrologic weathering may be a rapid process that deserves further investigation.

### Bioregenerative Life Support Systems

To recycle consumed nutrients from undesirable plant and human wastes, the use of bioregenerative life support systems (BLiSS) that decompose organic matter into inorganic nutrient streams will be used.^10^ Organic wastes (e.g., plant material, human wastes, etc.) generated by lunar and martian astronauts will necessitate recycling via a series of BLiSS bioreactors that result in effluent streams of water, inorganic nutrients, and metabolites of anerobic digestion, which include various volatile organic acids.^11^ A working BLiSS prototype at Kennedy Space Center (KSC), called the organic processing assembly (OPA), utilizes dual-stage anerobic bioreactors and membrane filtration^12,13^ to accomplish decomposition of organic matter, which is subsequently fed to a phototrophic membrane bioreactor^14^ for the complete oxidation of N species. The microbiome of OPA varies across reactors as a function of varying inputs and substrate decomposition.^15^ While OPA is a developing technology in both biological processing and technological command, analogous BLiSS, will be required to create a circular economy of nutrients in space.^16^ Because BLiSS produces nutrient streams that differ from those of inorganic nutrient solutions, investigating fundamental interactions of BLiSS effluent with regolith simulants will help guide future planning and determine uses of effluent for outposts on the Moon and Mars. In this study, effluent from the OPA at KSC was utilized, as it is a high-fidelity prototype of what may be produced in spaceflight and on lunar and martian outposts.

### Lunar and Martian Regolith

Lunar regolith is relatively homogeneous in elemental composition, with the major difference being the abundance of Ca. A useful classification refers to lunar samples as being “high-Ca” or “low-Ca” above or below 13.5 wt % CaO.^17^ Increasing abundance of nanophase Fe, along with decreasing grain size, is characteristic of more mature lunar regolith.^18^ Lunar samples exhibit low electrical conductivity (EC), though the electrical properties of lunar regolith are especially sensitive to thermal and atmospheric changes.^19,20^ In general, the chemical reactivity of lunar regolith to organic molecules is not well understood,^21^ but as aqueous and oxidizing environments are introduced to support plant life, it should be expected that lunar regolith will drastically change. For instance, exposure to water vapor increases the adsorption of N_2_ and Ar,^22^ while the crushing of regolith promotes the movement of trapped vaporous gases into solution.^23^ Lunar simulants have additionally demonstrated increased generation of reactive oxygen species (ROS) (e.g., H_2_O_2_ and OH^–^) in micromolar quantities when crushed and exposed to the atmosphere.^24^ Lunar regolith contains up to 70% agglutinates by volume,^25^ which are aggregates of mineral/lithic fragments fused together by rapid melting and cooling of glass after meteoroid impacts. Agglutinates are noted to have differing abundances of nanophase Fe^0^, with several hypotheses accounting for their incorporation into the lunar regolith.^26^

Martian astronauts will have greater accessibility to specific and diverse minerals for ISRU than lunar astronauts. For Mars, there is likely a semiactive hydrologic cycle of hydrated brines^27–30^ that may even support microbial life.^31^ Although glass is a more common feature of lunar regolith, in analog martian sites on Earth, glass-matrices have been identified as the possible profile-controlling minerals.^32^ Martian regolith is high in Fe metal oxides,^33^ chloride,^34^ and sulfate salts,^35^ including perchlorates (ClO_4_^–^) and other oxidants, such as hydrogen peroxide (H_2_O_2_), which occur at toxic levels for plants. These salts may limit bioavailability of various metals.^36^ Oxidized nitrogen species, simple organics, oxychlorine phases, and sulfates are widespread, with phyllosilicates and carbonates occurring in select Gale Crater materials, and it appears that geochemical conditions and organic C may have once been favorable for microbial life.^37,38^ Recent evidence from the Phoenix landing site quantified organic C at 83–1484 μg C g^–1^ and carbonates from 1.1 to 2.6 wt %.^39^ At Gale Crater, the 2007 Phoenix Mars Scout Mission’s Wet Chemistry Laboratory (WCL) conducted in situ experiments at the Phoenix landing site and found solutions were dominated by ClO_4_^–^, Mg^2+^, and Na^+^, at mM levels, with sub-mM concentrations of Ca^2+^, K^+^, and Cl^–^ and a pH of 7.7.^34^ The minimal leachable ions from surface samples had a corrected electrical conductivity (EC) ranging from 1370 to 1900 μS cm^–1^ at 25 °C. As the WCL extraction cell measured soil extracts at 1:25 (solid/solution), the reported EC values would likely be 5–25 times higher using traditional soil approaches (saturated paste extract; 1:1–1:5), indicating a highly saline soil that will need to be remediated prior to being used as a plant growth substrate, of which leaching with BLiSS solutions may be an effective management strategy.

### Overview

As BLiSS effluent streams will be an available resource for off-world outposts, the application of OPA’s effluent streams of the OPA to regolith simulants requires both a fundamental investigation of nutrient–regolith interactions and assessment of ISRU potential. The goal of the study was to determine if BLiSS effluent could be used to weather regoliths and provide plants with essential nutrients in the liquid phase. BLiSS effluent from the OPA at KSC, a half-strength Hoagland’s solution, and DI water were reacted with lunar (JSC-1A) and martian (MGS-1) simulants. Using batch experiments, the objectives were to investigate: (1) sorption to and dissolution of elements from the regolith; (2) elemental bonding of the regolith; (3) weathering of the regolith; and (4) mineralogical changes. It was hypothesized that due to the organic complexity of BLiSS effluent, there would be greater weathering compared to nutrient solution and water, and that lunar regolith simulant would be less reactive than the martian regolith due to its underlying minerology.

## Methods

### Materials

A lunar simulant (JSC-1A)^40,41^ and a martian simulant (MGS-1)^42^ were used in this study. The chemistry and micrology of the simulants are presented in Table 1. The reacting solution was the final effluent of the OPA BLiSS system at KSC, which was fed complex organic particulate artificial sewage (COPAS),^43^ a simulated waste, at a ratio of 50 g to 1 L of tap water. The artificial sewage was degraded into a primarily inorganic solution in an OPA BLiSS system and filtered at 0.22 μm. The resulting BLiSS filtered solution, called the effluent in this study, was used with an unadjusted pH of 7.0. To compare to an inorganic solution, a half-strength Hoagland’s solution^1^ was prepared using stock solutions and DI water and adjusted to pH 5.8 (Table 1), which is a common pH for spaceflight plant growth experiments.

**Table 1:** Chemistry and Mineralogy of the Lunar (JSC-1A) and Martian (MGS-1) Simulants Used in the Study

simulant	mineral		%	element	%
JSC-1A	glass-rich basalt	*n*Na_2_O·*n*CaO·*n*SiO_2_	49.3	Si	47.7
	Plagioclase	(Na,Ca)(Al,Si)AlSi_2_O_8_	37.1	Al	15.0
	olivine	(Mg,Fe)_2_SiO_4_	9.0	Ca	10.4
	Cr-spinel	(Mg, Fe^2+^)(Cr, Al, Fe^3+^)_2_O_4_	1.1	Mg	9.0
	Ti-magnetite	Fe^2+^(Fe^3+^,Ti)_2_O_4_	0.4	Fe	10.8
	K-silicate	K_2_O_3_Si	1.4	Na	2.7
	sulfide	S^2–^	1.0	Ti	1.6
	albite	NaAlSi_3_O_8_	0.3	K	0.8
	quartz	SiO_2_	0.2	P	0.7
	chlorite	ClO_2_	0.1	Mn	0.2
				Cr	0.0
				LOI	0.7
				total	99.7
MGS-1	Anorthosite	CaAl_2_Si_2_O_8_	27.1	Si	43.9
	glass-rich basalt	*n*Na_2_O^•^ *n*CaO^•^ *n*SiO_2_	22.9	Al	12.8
	Bronzite	(Mg,Fe)_2_[Si_2_O_6_]	20.3	Fe	10.6
	Olivine	(Mg,Fe)_2_SiO_4_	13.7	Mg	14.8
	Epsomite	MgSO_4_^•^ *n*H_2_O	4.0	Ca	7.9
	Ferrihydrite	Fe_2_O_3_^•^ *n*H_2_O	3.5	Na	1.5
	hydrated silica	SiO_2_^•^ *n*H_2_O	3.0	Ti	0.5
	Magnetite	Fe_3_O_4_	1.9	K	0.3
	Gypsum	CaSO_4_	1.7	P	0.2
	Siderite	FeCO_3_	1.4	Mn	0.1
	Hematite	Fe_2_O_3_	0.5	LOI	4.9
				total	97.5

### Experimentation

For batch experiments, 0.5 g of simulant (JSC-1A or MGS-1) was added to 50 mL centrifuge tubes, followed by 25 mL of reacting solution (effluent, half-strength Hoagland’s solution, or DI water). In addition to the batch experiment, an isotherm study was conducted with the same solid/solution ratio (1:50) used for the batch experiment, but dilutions of reacting solutions were made with DI water to 100× (dilution), 80×, 60×, 40×, 20×, 10×, 5×, 2×, 1× (no dilution).

Samples were reacted on an orbital shaker at 60 rpm for 24 h at room temperature (22 °C). For liquid analysis, the samples were centrifuged, and the supernatant was decanted into separate containers and stored at 4 °C until analysis. For solid analysis, the simulants were rinsed with 10 mL of DI water, centrifuged twice to remove exogenous solution, and then dried at 40 °C overnight prior to analysis. For the batch experiment, there were three experimental replicates for each solution × substrate combination, and for the isotherm experiment, samples were duplicated.

### Analyses

After the 24 h batch experiment, the pH and electrical conductivity (EC) of the reacting solutions were measured to assess changes to solution chemistry after weathering. Solutions were acidified to 1% HNO_3_ and analyzed via inductively coupled plasma-optical emission spectroscopy (ICP-OES) (Thermo Scientific, iCAP 7000) in duplicate. Ion chromatography was used to analyze the ions without any sample preparation. Substrate samples were Au sputter-coated (Denton Vacuum, Desk IV) and mounted prior to scanning electron microscope-energy dispersive X-ray spectroscopy (SEM-EDS) (Jeol, JSM-IT800). X-ray photoelectron spectroscopy (XPS) (Thermo Scientific, Nexsa G2) was used on substrates to identify elemental bonding associations by using Qtegra software. XPS data were processed in the Avantage Data System. X-ray diffraction (XRD) (Malvern Panalytical, Empyrean) was used to assess mineralogical changes to substrates and was performed using Bragg–Brentano mode. The diffraction data were recorded from 15 to 70 two-theta (2θ) degrees. HighScore curve fitting software was used to process the diffraction pattern and compared to the PDF-4+2023 International Center of Diffraction Database (ICDD).

The adsorption capacity (*q*_e_) of elements in the reacting solution at equilibrium was determined using *q*_e_ = (*C*_i_ – *C*_e_)/*W* × *V*, where *q*_e_ represents the sorption capacity (μmol g^–1^), *C*_i_ and *C*_e_ represent the initial and equilibrium concentrations (μM) of the adsorbate, and *V* and *W* stand for solution volume (0.25 L) and mass (0.5 g) of the adsorbent, respectively. Adsorption isotherms (Langmuir and Freundlich) were applied to explain the equilibrium adsorption characteristics if a *R* ^2^ > 50 was obtained. The Langmuir’s isotherm was represented as *q*_e_ = (*q*_max_ *K*_L_ *C*_e_)/(1 + *K*_L_ *C*_e_). The Langmuir’s isotherm was transformed into its linear form, as represented by 1/*q*_e_ = 1/(*K*_L_ *q*_max_) × 1/*C*_e_ + 1/*q*_max_, where *q*_max_ represents the maximum adsorption capacity (μmol g^–1^) and K_L_ (L μmol^–1^) is the Langmuir’s isotherm constant that represents the binding affinity between the element and the regolith simulant. The separation factor (*R*_L_) was calculated using *R*_L_ = 1/(1 + *C*_i_ × *K*_L_), where *R*_L_ is the dimensionless Langmuir constant, which indicates the adsorption possibility that is either favorable (0 < RL < 1), unfavorable (RL > 1), linear (RL = 1), or irreversible (RL = 0). The Freundlich’s isotherm was represented as *q*_e_ = *K*_f_ *C*_e_^1/*n*^. The linear form of Freundlich’s isotherm was used as Log *q*_e_ = Log *K*_f_ + 1/*n* Log *C*_e_, where *K*_f_ is the distribution coefficient used to measure the adsorption capacity and 1/*n* is the adsorption intensity. The value of 1/*n* demonstrates the adsorption process as either favorable (0.1 < 1/*n* < 1) or unfavorable (1/*n* > 1).

## Results

### Solution Characterization

Reacting solutions were characterized prior to the batch experiment (Table 2). Notable differences between solutions were the lower concentrations of Ca, Fe, K, S, Cl, NO_3_^–^, PO_4_^3–^, and SO_4_^2–^ and increased concentrations of Na, P, NH_4_^+^, and NO_2_^–^ in effluent compared to half-strength Hoagland’s solution. The dominant elements in the BLiSS effluent were N > Cl > Na > K > P > Mg, Ca > S. Additionally, the EC of the effluent was nearly three times greater than Hoagland’s.

**Table 2:** Composition and Properties of Reacting Solutions^a^

	half-strength Hoagland’s		effluent	
analyte	mg L^–1^	μM	mg L^–1^	μM
Elements				
Al	ND	ND	0.1	4.7
B	0.1	8.6	0.2	14.9
Ca	97	2420	28	690
Cr	0.1	1.0	ND	0.1
Cu	ND	0.6	ND	0.1
Fe	2.1	37	ND	0.3
K	172	4397	128	3268
Mg	29	1211	28	1135
Mn	0.2	4.5	0.0	0.1
Mo	<0.1	<0.1	0.0	0.0
Na	1.0	44	154	6687
Ni	ND	ND	ND	ND
P	16.2	524	64	2062
S	32	993	17	520
Zn	0.1	1.2	ND	ND
Ions				
ammonium (NH_4_^+^)	5.4	297.1	292.8	16,230.9
chloride (Cl^–^)	54.8	1545.7	214.8	6058.0
nitrate (NO_3_^–^)	439.3	7085.6	43.3	699.0
nitrite (NO_2_^–^)	11.9	257.7	858.8	18,668.4
phosphate (PO_4_^–^)	52.1	549.1	116.4	1225.5
sulfate (SO_4_^–^)	87.0	905.5	37.1	386.1
pH	5.8		7.0	
EC (μS cm^–1^)	713		2070	

a ND indicates not detectable.

### Solution pH and Conductivity

After the 24 h batch experiment, the pH of both solutions increased. The Hoagland’s solution, which was slightly acidic, trended toward becoming neutral, whereas the effluent, which was neutral, became slightly more alkaline. Hoagland’s reacting solution increased from 5.8 to 6.64 or 6.67 in JSC-1 and MGS-1, respectively, and effluent increased from 7 to 7.47 or 7.27 in JSC-1 and MGS-1, respectively. The EC also increased in both solutions, but there was a greater increase from MGS-1 (Table 2), indicating more dissolution of MGS-1 than JSC-1 (Table 3). The EC values of solutions are considered slightly saline after the 24 h shaking experiment.

**Table 3:** Reacting Solution pH and EC after 24 h Batch Experiment^a^

	JSC-1A		MGS-1	
measurement	Hoagland’s	effluent	Hoagland’s	effluent
pH	6.64	7.47	6.74	7.27
EC (μS cm^–1^)	853	2487	1299	2870

a Results are an average of triplicate.

### Sorption to Regolith

The concentrations of elements in the two solutions were reactive at various dilutions to lunar (Figure 1) or martian (Figure 2) substrates, indicating competitive sorption or regulated dissolution. Phosphorus, K, and Zn were the only elements observed to exhibit consistent sorption across dilutions that could be modeled with either Langmuir or Freundlich’s isotherms (e.g., *R* ^2^ > 0.50) (Figure 3a; Table 4). Phosphorus from both solutions sorbed to MGS-1, but neither solution sorbed to JSC-1A. Phosphorus sorption was best fit with the Langmuir isotherm model, indicating potential monolayer sorption. Phosphorus sorption to MGS-1 was substantial with a maximum sorption capacity (*q*_max_) of 7.103 and 17.685 μmol g^–1^ for Hoagland’s and effluent, respectively, and a higher binding affinity (K_L_) for Hoagland’s of 1.578 than effluent of 0.237 L μmol^–1^. Potassium sorption occurred only with Hoagland’s solution in both JSC-1A and MGS-1 (Figure 3b). Freundlich was a better fit for K reactivity to both substrates, indicating potential multilayer sorption at specific sites, albeit “unfavorable” binding strengths (i.e., 1/*n* < 0.1). Zinc was sorbed to MGS-1 but only from Hoagland’s and was also fit to a Freundlich isotherm (Figure 3c), again showing “unfavorable” binding strengths (i.e., 1/*n* < 0.1).

**Figure 1:**
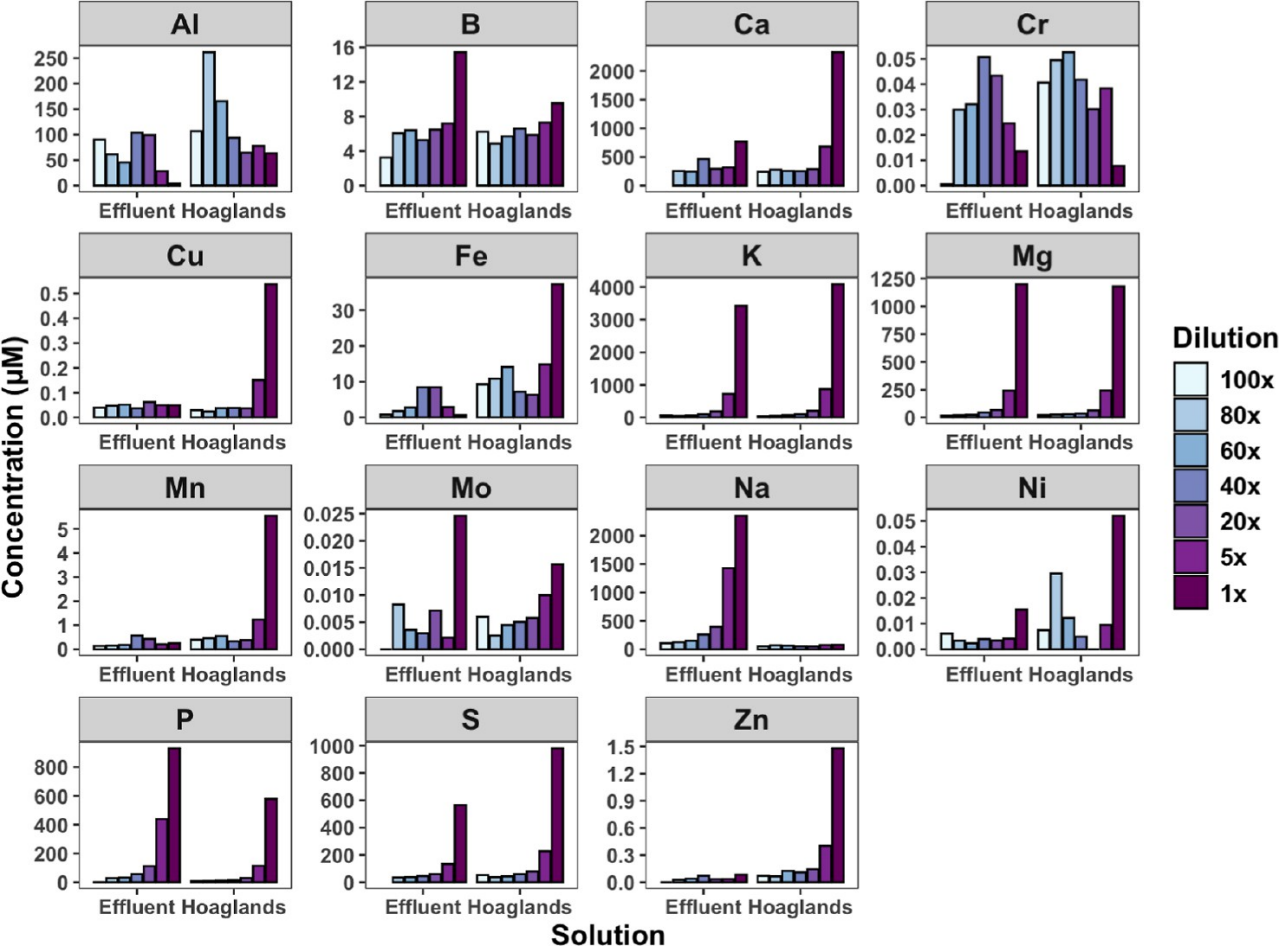
Equilibrium concentration of reacting solutions with lunar simulant JSC-1A across dilutions of two solutions, Hoagland’s and BLiSS effluent.

**Figure 2:**
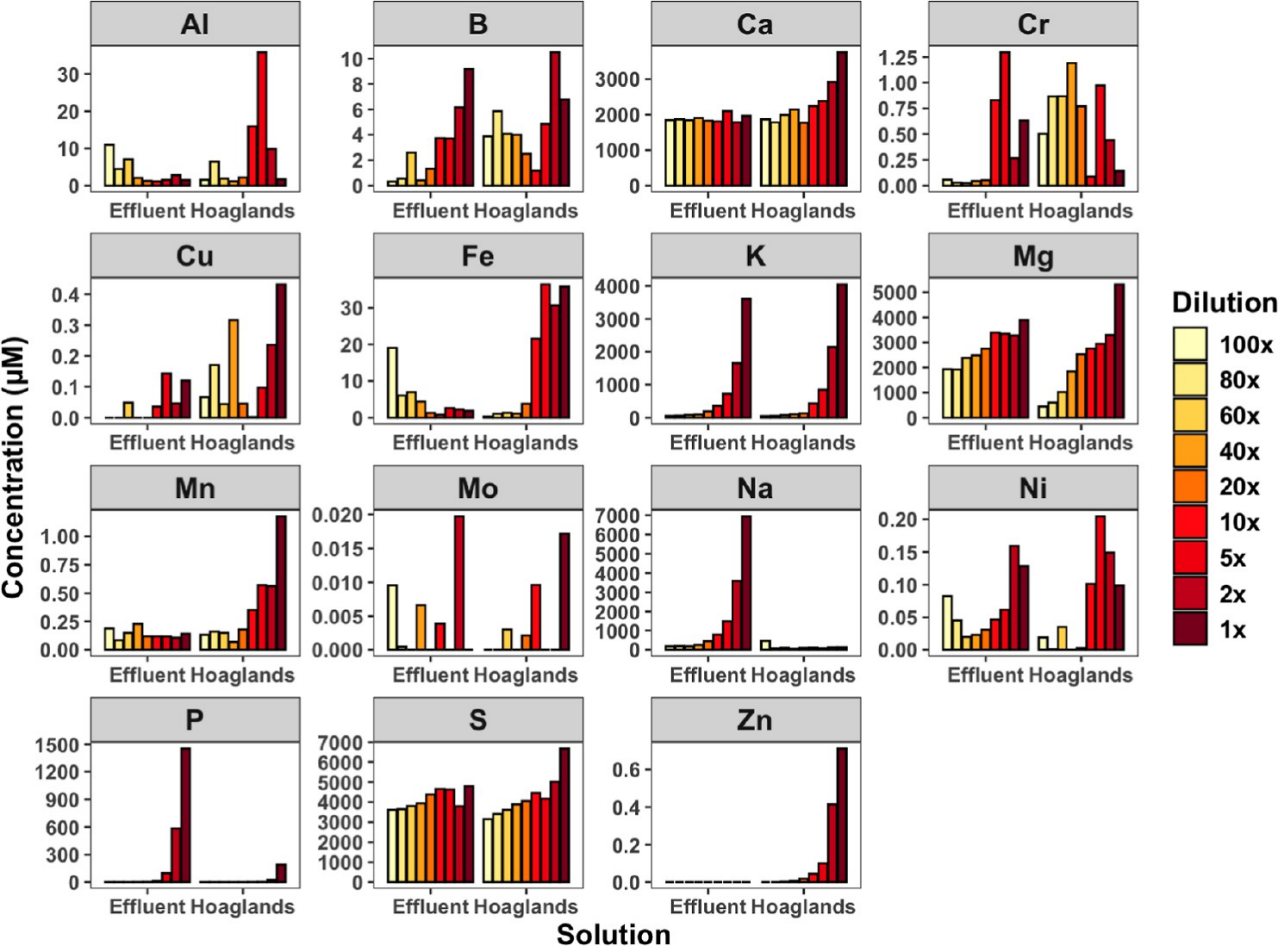
Equilibrium concentration of reacting solutions with martian simulant MGS-1 across dilutions of two solutions, Hoagland’s and BLiSS effluent.

**Figure 3:**
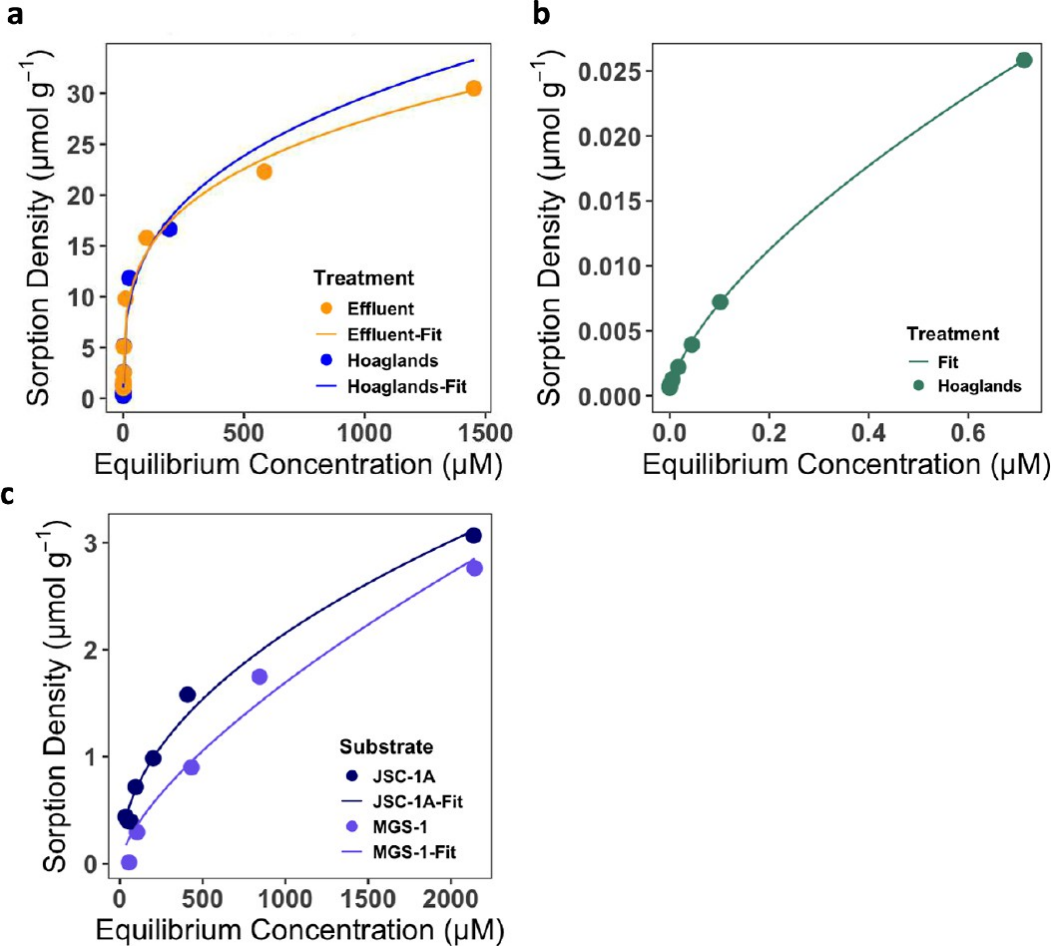
Adsorption isotherms of specific elements that could be modeled with *R* ^2^ > 0.5 in the multielement solutions used in experimentation, showing reactivity of (a) phosphorus, (b) zinc, and (c) potassium with points representing experimental data and lines representing isotherms. Substrates consisted of either lunar (JSC-1A) or martian (MGS-1) simulants, and solutions consisted of either Hoagland’s or BLiSS effluent. Results are the average of duplicates.

**Table 4:** Isotherm Parameters of the Sorption of Phosphorus, Potassium, and Zinc to Lunar (JSC-1A) and Martian (MGS-1) Simulants

			JSC-1A		MGS-1	
element	isotherm	parameters	Hoagland’s	effluent	Hoagland’s	effluent
phosphorus	Langmuir	*q*_max_ (μmol g^–1^)	--	--	7.103	17.685
		*K*_L_ (L μmol ^–1^)	--	--	1.578	0.237
		*R*_L_	--	--	0.388	0.809
		*R* ^2^	--	--	0.996	0.992
potassium	Freundlich	*K*_f_	0.019	--	0.001	--
		1/*n*	0.777	--	1.381	--
		*R* ^2^	0.966	--	0.890	--
zinc	Freundlich	*K*_f_	200.586	--	--	--
		1/*n*	1.593	--	--	--
		*R* ^2^	0.953	--	--	--

### Dissolution of Regolith into Solution

In general, the weathering and subsequent dissolution of JSC-1A were far less than those of MGS-1. Dissolution of JSC-1A is characterized by the release of metals such as Al, Cu, Mn, Ni, and Zn. Fe was released only into DI water, but not Hoagland’s or effluent solution. On the other hand, the dissolution of MGS-1 led to the drastic release of S, the alkali/alkaline metals Ca, Mg, and Na, and other metal release of Mo and Ni. To investigate which minerals had weathered, minerals were investigated with XRD; however, no changes to mineralogy were identified. At full concentration of solutions, the sorption density of each element is included in Figure 4. A summary of the sorption density at full concentration is included in Table 5.

**Figure 4:**
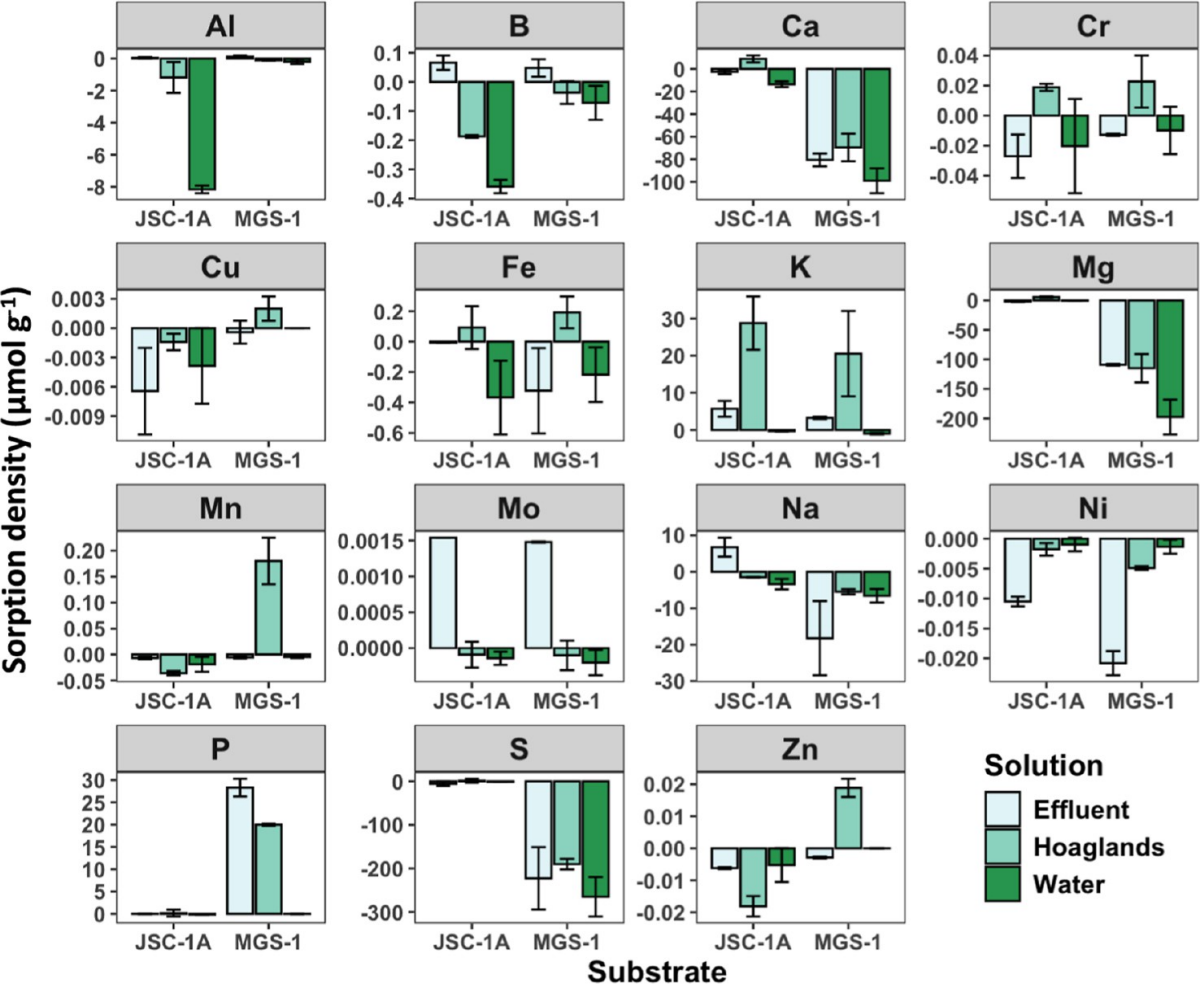
Sorption density of reacting solutions with lunar (JSC-1A) and martian (MGS-1) simulants. Positive values indicate net sorption, while negative values indicate net dissolution. Data are displayed as average ±SD.

**Table 5:** Summary of Sorption or Dissolution of Lunar Simulant JSC-1A and Martian Simulant MGS-1 after 24 h Reaction Time with Bioregenerative Life Support System (BLiSS) Effluent and Hoagland’s Nutrient Solution

	JSC-1A		MGS-1	
element	Hoagland’s	effluent	Hoagland’s	effluent
Al	--	dissolution	--	--
B	sorption	dissolution	sorption	dissolution
Ca	dissolution	sorption	dissolution	dissolution
Cr	dissolution	sorption	dissolution	sorption
Cu	dissolution	dissolution	--	sorption
Fe	--	--	dissolution	sorption
K	sorption	sorption	sorption	sorption
Mg	--	sorption	dissolution	dissolution
Mn	--	dissolution	--	sorption
Mo	sorption	--	sorption	--
Na	sorption	dissolution	dissolution	dissolution
Ni	dissolution	dissolution	dissolution	dissolution
P	--	--	sorption	sorption
S	dissolution	--	dissolution	dissolution
Zn	dissolution	dissolution	dissolution	sorption

### Weathering of Substrates

Visual observations of the substrates after the batch experiment revealed weathering to the minerals. In JSC-1A, the sharp mineral edges appeared to have some rounded-off features that reduced the overall sharpness of corners and edges (Figure 5); however, a quantitative analysis was not available to indicate if mineral sharpness was statistically reduced. In general, the overall particle size was not substantially reduced in a survey of all of the minerals. Notable artifacts of the weathering process include holes in the mineral faces where small particles collapsed and the appearance of webbing patterns in the anorthosite fraction of JSC-1A. Unfortunately, EDS was not able to detect any sorbed elements in these features (Figure 6). Because the detection limits of EDS are ∼1000 mg kg^–1^, it is reasonable that no sorbed features were detected, given that measured sorption densities after batch experimentation were lower than EDS detection limits. Further, the probed volume at a given electron energy is below the surface, whereas adsorbents are likely only located on the mineral surface.

**Figure 5:**
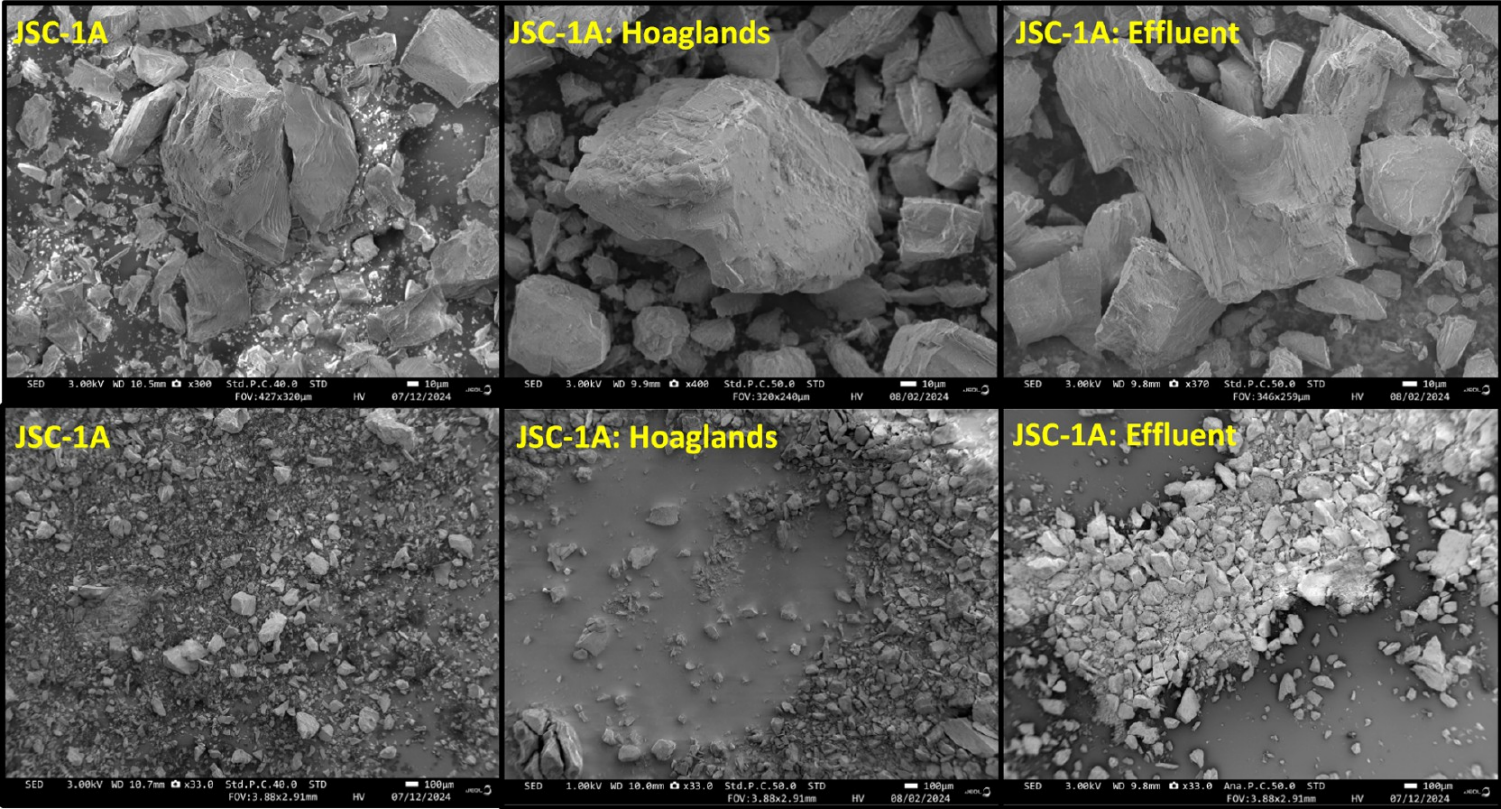
Lunar simulant JSC-1A imaged with scanning electron microscope (SEM). The electron beam energy ranged from 1 to 3 kV. The Hoagland’s and effluent images are after the 24 h batch experiment.

**Figure 6:**
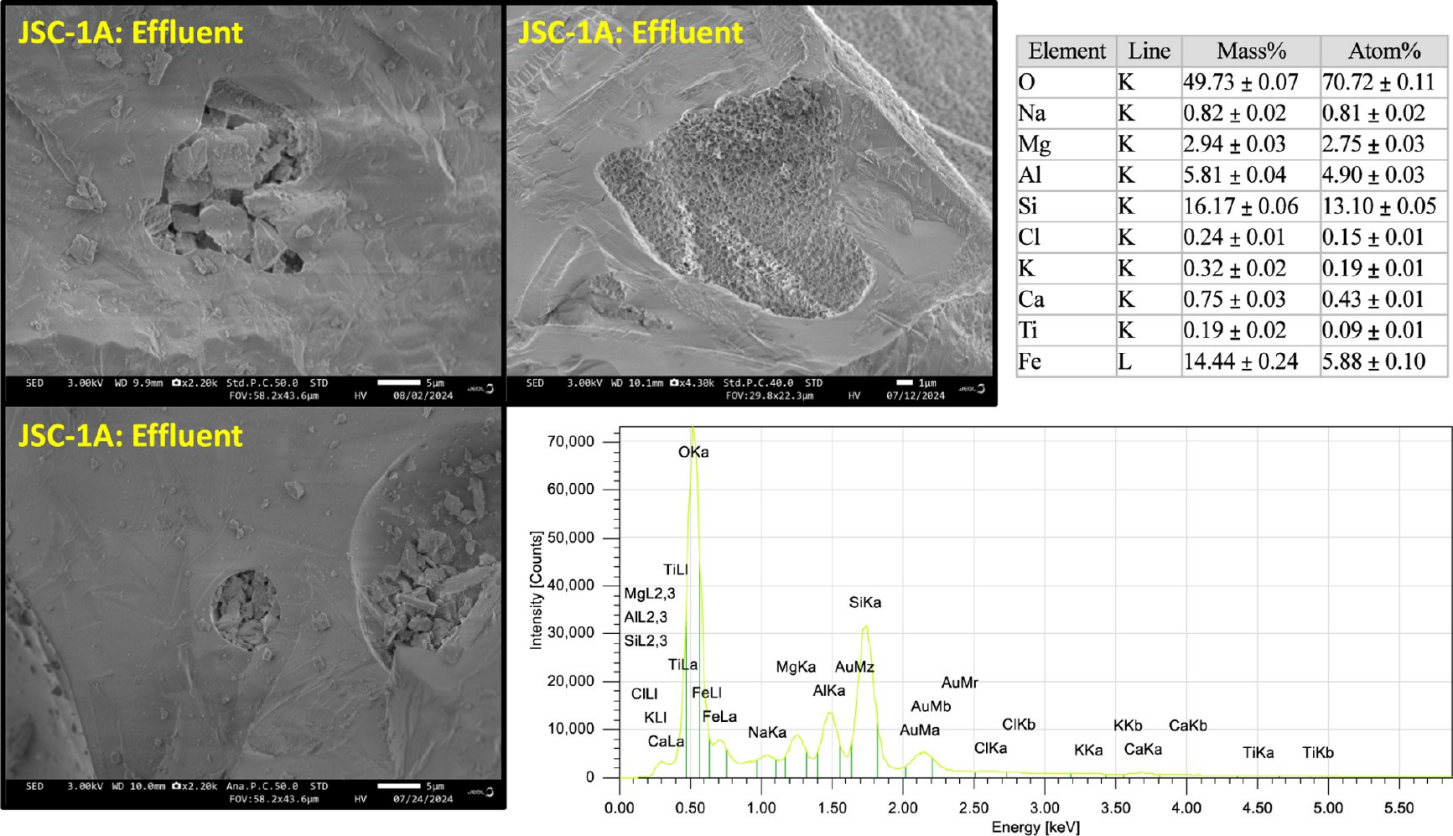
Scanning electron microscope (SEM) image of erosion spots on lunar simulant JSC-1A after batch experiment. Electron dispersive spectroscopy (EDS) was used to investigate the elemental abundance of erosion regions and was set to 30 keV.

In MGS-1, there appeared to be a reduction in particle size in a survey overlooking the samples (Figure 7). Nano/microsized particles were clearly seen to adhere to MGS-1 after the weathering event; again, EDS did not detect any of the sorbed elements in these features (Figure 8). In addition, diatoms were found in all MGS-1 samples (as stated by the manufacturer^42^). For MGS-1, electrical charge buildup made SEM imaging more difficult than that of JSC-1A.

**Figure 7:**
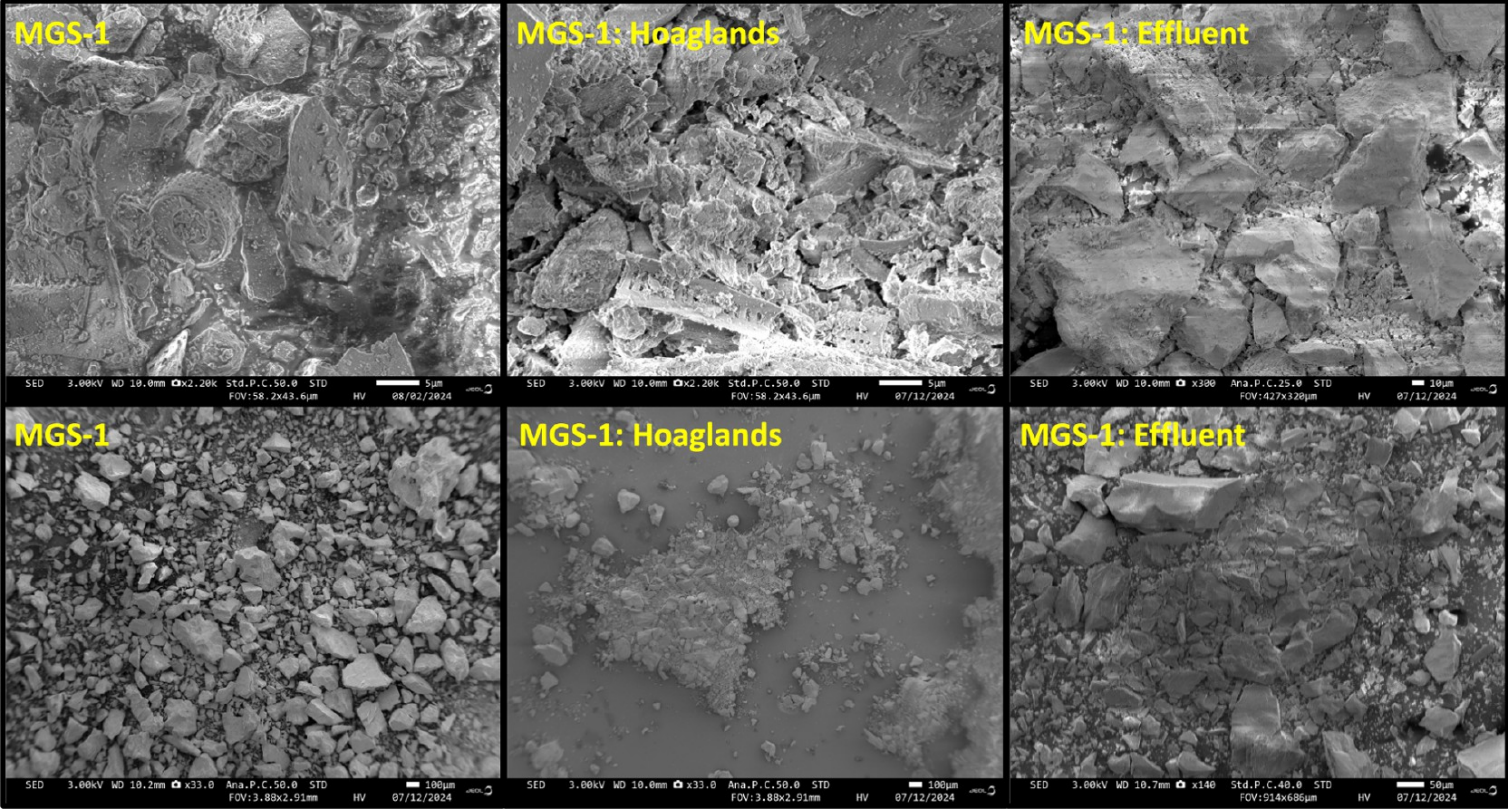
Mars simulant MGS-1 imaged with a scanning electron microscope (SEM). The electron beam energy ranged from 1 to 3 kV. The magnification factor varies slightly in the left and middle images from the right images of effluent. The Hoagland’s and effluent images are after the 24 h batch experiment.

**Figure 8:**
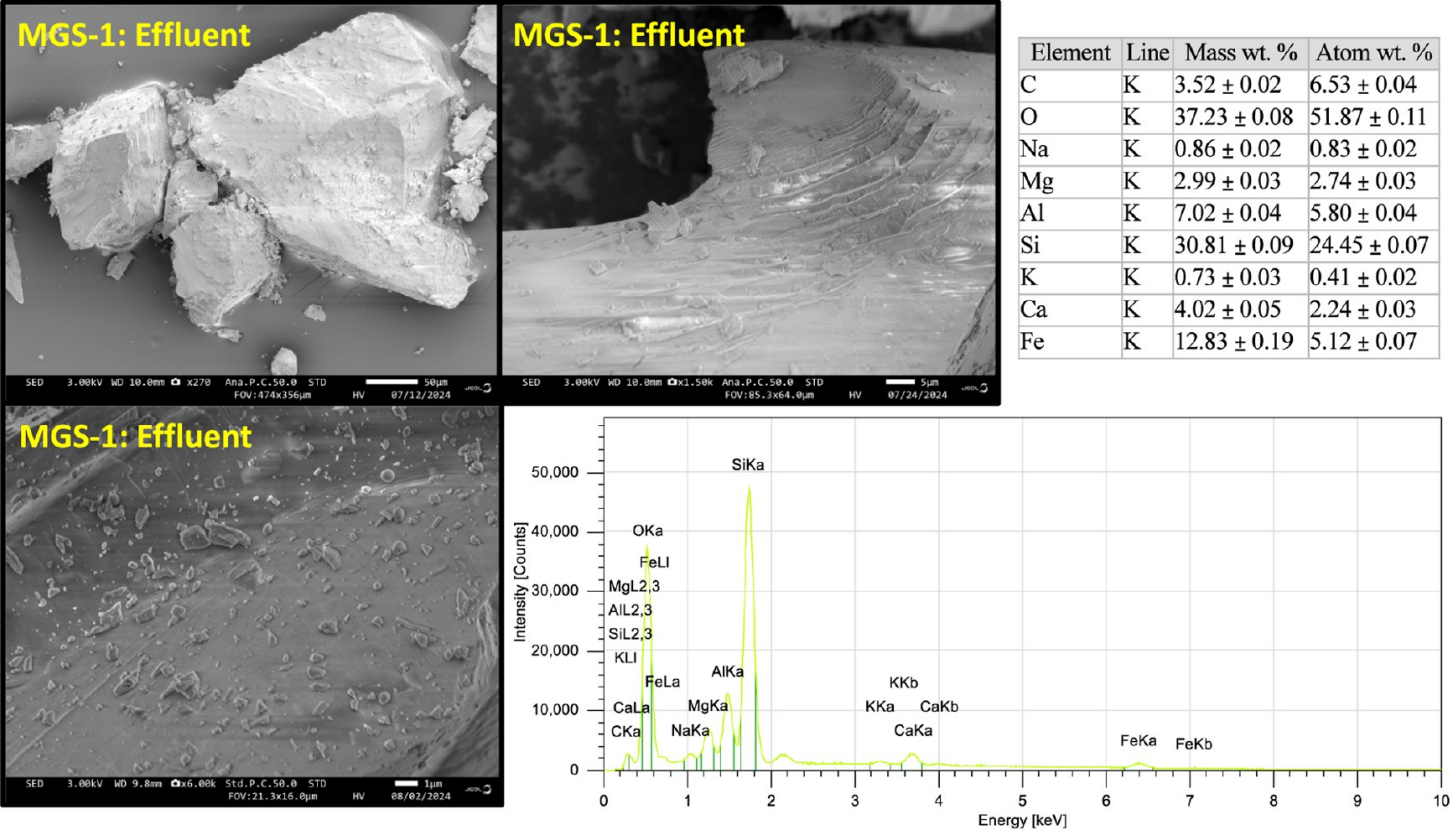
Scanning electron microscope (SEM) image of nanoparticles adhering to martian simulant MGS-1 after batch experiment. Electron dispersive spectroscopy (EDS) was used to investigate the elemental abundance of adhered nanoparticles and was set to 30 keV.

### Chemical Interaction

The elements C, N, P, and Ca were investigated with high-resolution XPS. For C, a major peak at ∼286 eV was observed in all substrates and solutions that corresponded to C–O bonding. Hoagland’s and effluent did not affect the XPS spectra significantly in JSC-1A; however, in MGS-1, there was a notable increase in the Hoagland’s and effluent-treated regolith at ∼294 eV, which corresponds to CF_3_ (Figure 9). Because F was not quantified, it is difficult to know if F was in differing concentrations between solutions. Hoagland’s was made from DI water and effluent processes a mixture of fecal simulant and tap water; it is unlikely that F was greater in Hoagland’s.

**Figure 9:**
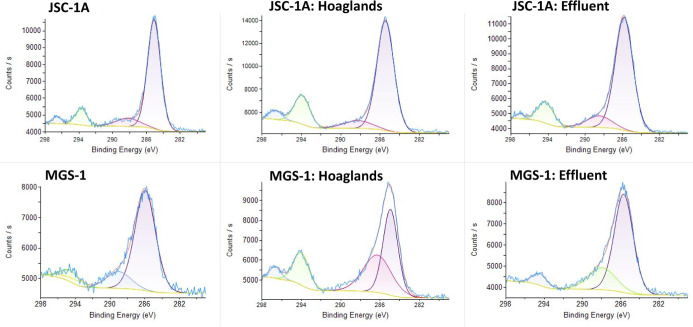
Carbon (C 1s) peaks of lunar (JSC-1A) and martian (MGS-1) simulants as unaltered materials and after batch experiment using XPS.

For N, the addition of Hoagland’s and effluent solutions resulted in a peak at ∼400–401 eV, which corresponds to C–NH_2_ (∼400 eV), N–(C=O) (400.3 eV), (C=O)–N–(C=O), or NSi_2_O (399.9 eV) in JSC-1A and MGS-1 (Figure 10). A large NH_4_^+^ abundance in effluent could account for C–NH_2_ peak; however, this is unlikely to be the peak as the only source of N in Hoagland’s was NO_3_^–^ (expected at 407.2 eV).

**Figure 10:**
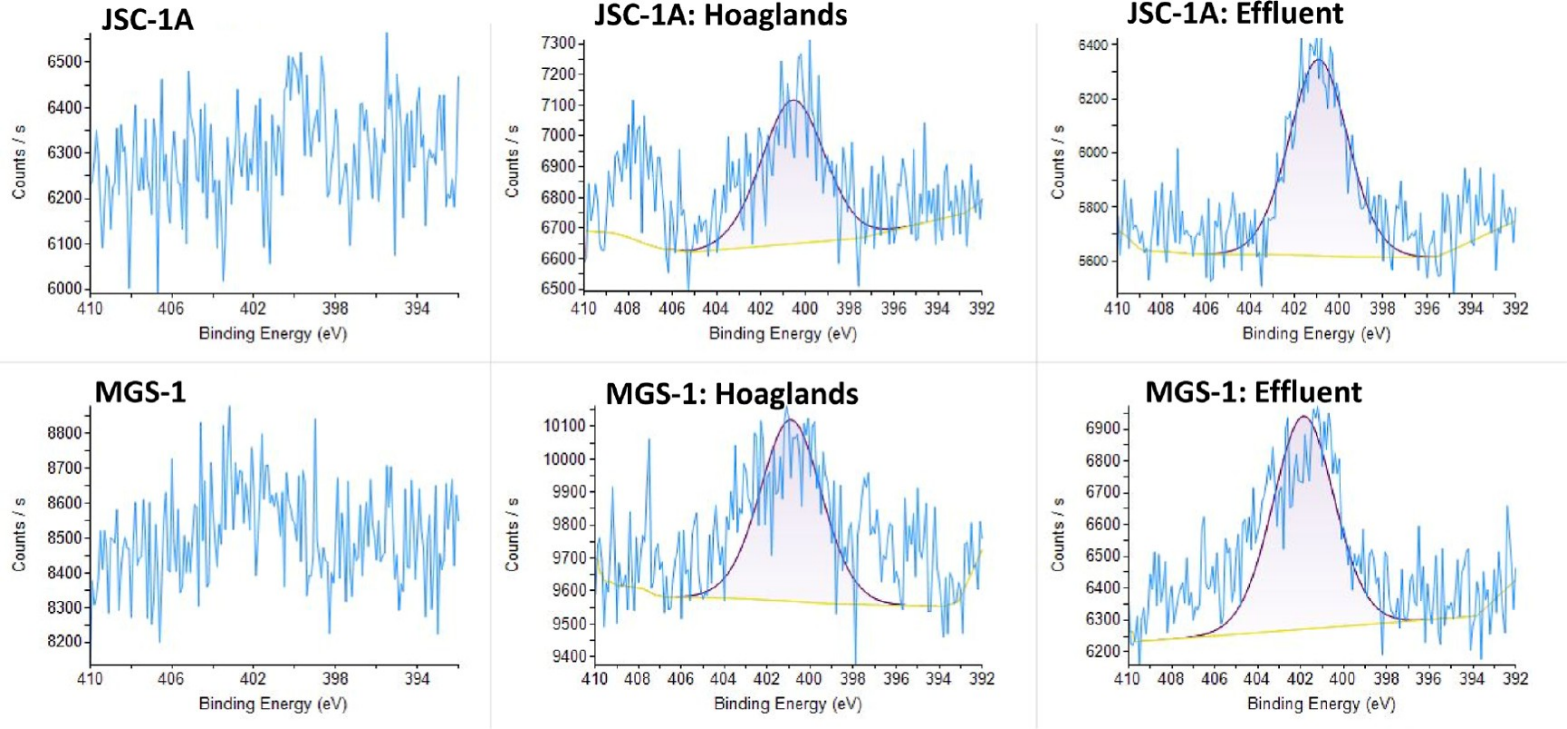
Nitrogen (N 1s) peaks of lunar (JSC-1A) and martian (MGS-1) simulants as unaltered materials and after batch experiment using XPS.

For P, clear peaks were indicative of metal phosphates (∼133 eV; Figure 11). For Ca, there were no differences in JSC-1A; however, MGS-1 had a peak at ∼348 eV, likely corresponding to Ca_3_(PO_4_)_2_ (Figure 12). In addition to the described elements, K, Al, Fe, and O were investigated, but no changes in the chemical states were observed with the experimental treatments.

**Figure 11:**
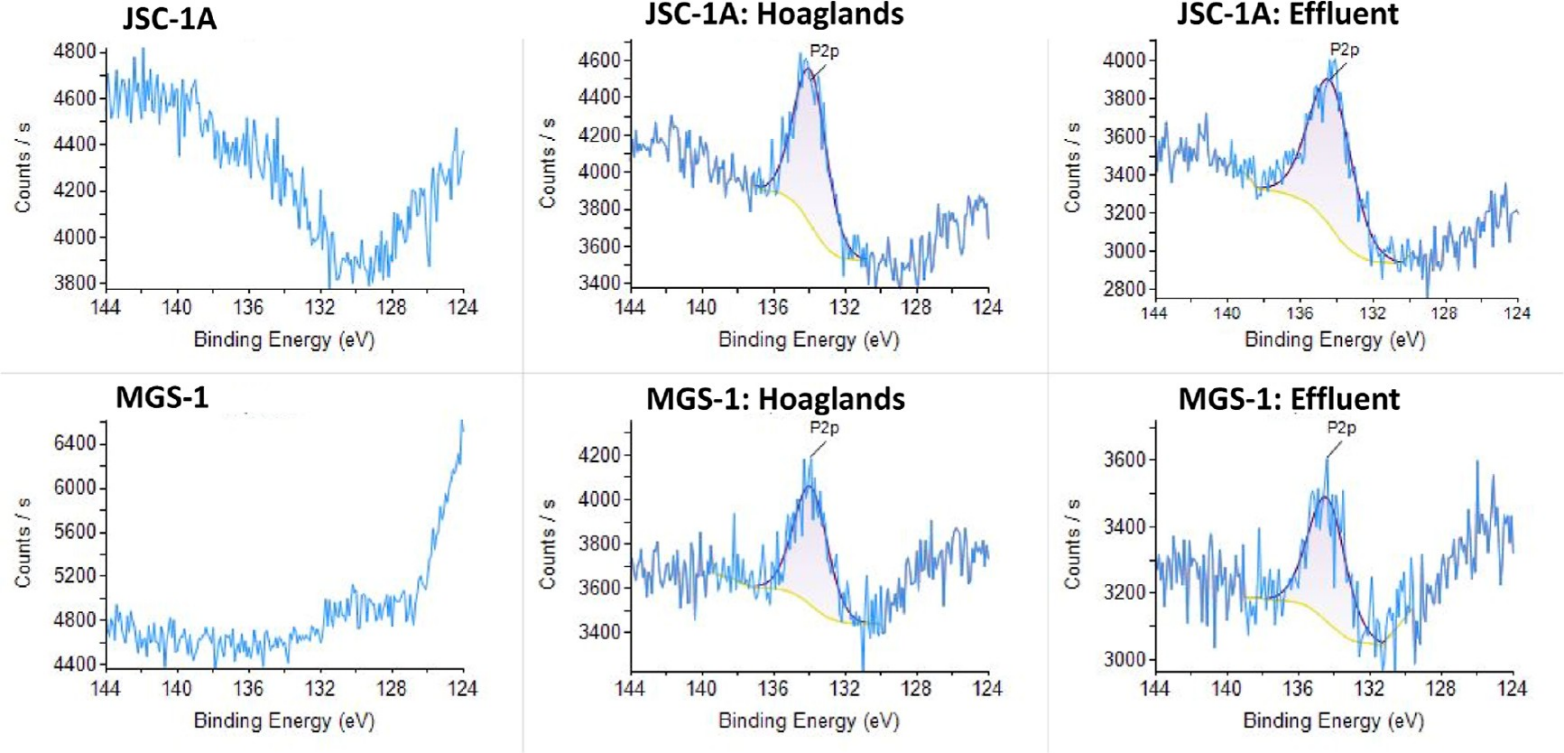
Phosphorus (P 2p) peaks of lunar (JSC-1A) and martian (MGS-1) simulants as unaltered materials and after batch experiment using XPS.

**Figure 12:**
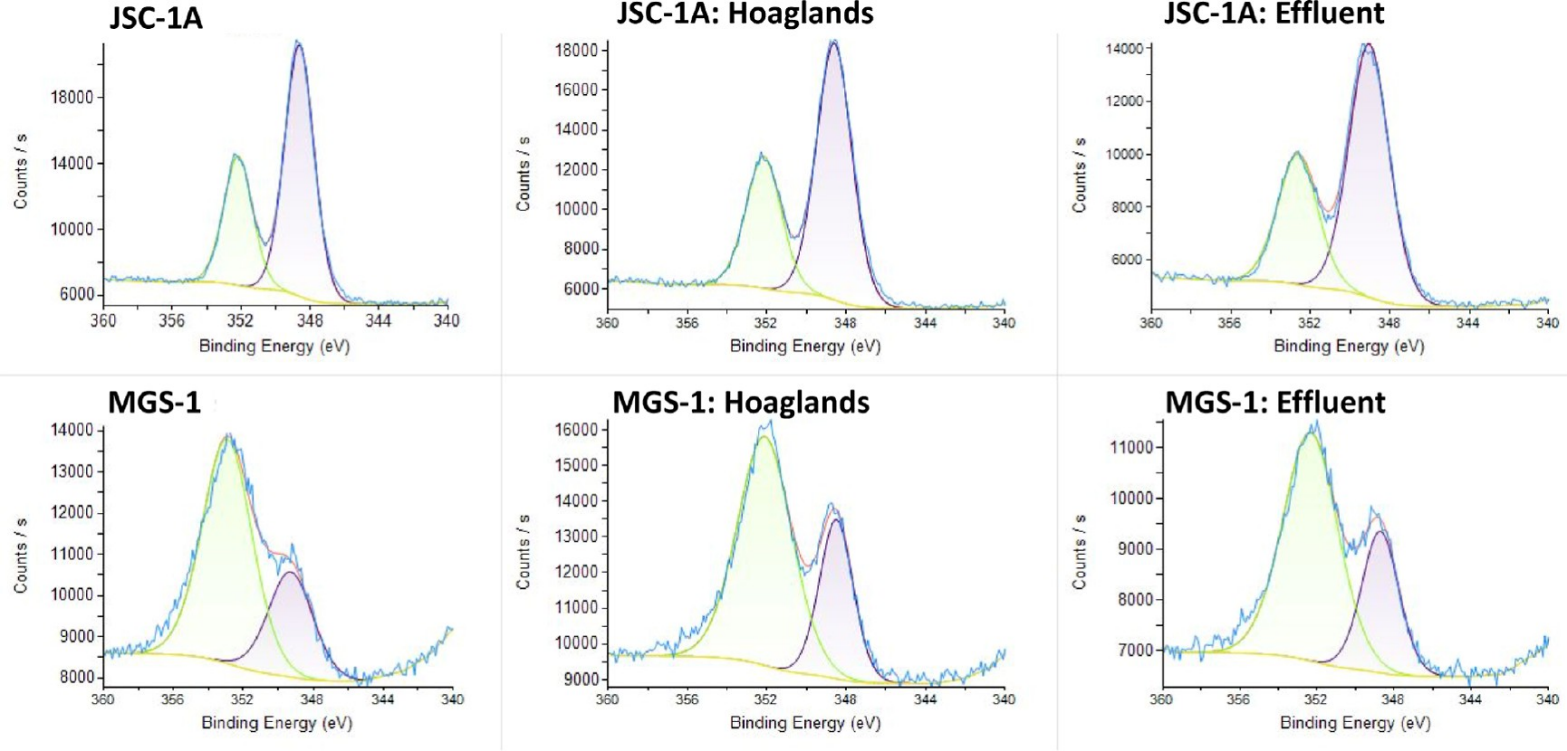
Calcium double peaklets (2p3/2 and 2p1/2) of lunar (JSC-1A) and martian (MGS-1) simulants as unaltered materials and after batch experiment using XPS.

## Discussion

The application of the BLiSS effluent to lunar and martian simulants resulted in variable dissolution of certain elements and sorption of P, K, and Zn, with minor sorption of other elements depending on the solution provided. It is clear that alteration of planetary materials will occur when hydrologic weathering is introduced, likely as a result of the relatively weatherable basaltic parent material present on both the Moon and Mars. Mineral weathering by BLiSS streams presents both potential for ISRU and enhancement of the regolith for subsequent plant growth in the altered minerals. As an example, scaling to 1 kg of regolith exhibits vast potential to supply major plant essential nutrients to BLiSS solution (Table 6). To date, there have been few experimental studies that evaluate how regolith simulants respond to hydrologic weathering and even fewer studies that evaluate if these regolith-weathered elements are plant available. This study provides evidence of the variable reactions of regolith across water, inorganic nutrient solutions, and high-fidelity BLiSS effluent and expands the use of regolith for ISRU applications.

**Table 6:** Weathering Regolith with BLiSS Solutions Leads to Element Extraction at the Scale of Inorganic Nutrient Solutions^a^

element	half-strength Hoagland’s (μM)	1 kg JSC-1A (μM)	1 kg MGS-1 (μM)
Al	0	–43	–117
B	9	–66	–48
Ca	2420	2487	80,609
Cr	1	27	13
Cu	1	6	0
Fe	37	7	323
K	4397	–5658	–3247
Mg	1211	1818	109,064
Mn	5	7	6
Mo	<0.1	–2	–1
Na	44	–6741	18,236
Ni	0	10	21
P	524	–3	–28,308
S	993	5569	222,752
Zn	1	6	3

a For instance, weathering 1 kg of lunar or martian regolith simulant in 1 L of BLiSS solution for 24 h led to net dissolution of the mineral with limited sorption of a few elements. Positive values indicate dissolution of regolith into solution, while negative values indicate elements removed from solution as sorption to regolith.

As for linking the results of simulants to those of genuine lunar and martian soils, it is important to consider that genuine soils are exceptionally heterogeneous in chemical speciation and mineralogy, while simulants are broadly representative. A difficult area to reproduce in simulants is the redox state, which on the Moon is highly reduced, whereas Mars is highly oxidized. For lunar surface soils, being highly reduced and having a large fraction of nanophase Fe when introduced to water will likely lead to the generation of a highly alkaline solution and potential reactive oxygen species (ROS) such as hydroxy radicals (OH*), which are strong oxidizing agents in biology. Furthermore, the reactivity of lunar agglutinates to aqueous streams remains unknown, and agglutinates are not well represented in the JSC-1A simulant. Despite their low abundance (∼0.5%), JSC-1A contains minor and trace nonlunar components (carbonates, sulfates, nitrates, clays, etc.) that may be highly relevant to this study and disproportionately affect the results despite their low abundance, as they may be more reactive than genuine lunar components. For the martian simulant MGS-1, the epsomite and gypsum used in the simulant represent highly soluble chemicals that will readily dissolve when they interact with an aqueous stream. For instance, Table 6 represents strong S, Mg, and Ca signatures for MGS-1 that derive from epsomite and gypsum. The high solubility of individual components in the MGS-1 simulant somewhat reflects the water extractible ions confirmed by the Phoenix Lander’s WCL, where a high abundance of water extractable salts was detected at the measured location.^34^ It is therefore likely that on Mars, a highly soluble portion of the surface minerals may be reactive toward BLiSS streams even within the short-term window used for this experimentation (24 h). It is important to note that chlorates and perchlorates were not incorporated into the simulants used in this study for safety reasons; however, they are widespread on Mars and are highly soluble as well and should be expected to be dissolved into solution, albeit the interaction of organic BLiSS streams with perchlorates remains outstanding.

Minerals common to lunar and martian surfaces are among the least stable in terrestrial soils and sediments,^3^ especially as they lack hydrologic weathering as on Earth. Thus, the rapid weathering of simulants in this study released various elements into the solution. For BLiSS-treated regolith, lunar simulant JSC-1A supplied Al, B, Cu, Na, Ni, Mn, and Zn consistent to other studies of lunar regolith simulants,^44^ whereas martian simulant MGS-1 supplied Al, B, Ca, Mg, Na, Ni, S, and Zn. While Al and Na are not considered plant essential elements, they can provide benefits to plants when provided in submacro quantities.^45^

The BLiSS stream used here lacked various plant essential mineral elements such as Cu, Fe, Mn, S, and Zn compared to the half-strength Hoagland’s solution. As most of these elements were present in the waste stream input to the BLiSS, it is likely they precipitated as various mineral phases^46^ or sorbed to minerals within the BLiSS. Anerobic bioreactors and their associated membrane filtration units encounter fouling as Ca-, P-, and Mg-containing mineral (i.e., hydroxyapatite, dolomite, and struvite) precipitate,^47^ or as extracellular polymeric substances (EPS) accumulate from biological activity.^48^ Minerals such as calcium phosphate are well-known to trap metal ions.^49^ Thus, waste streams provided to BLiSS may not have full recovery of mineral elements. For instance, while there was no Zn detected in the final effluent, Zn was supplied to OPA at a concentration of approximately 75 mg kg^–1^ ^43^ and a rate of 9 mg Zn day^–1^, and Zn readily sorbs to calcium phosphate minerals.^50^ In lunar and martian colonies, the loss of such mineral elements will impede the productivity of the effluent for such purposes as being used as fertigation for plant growth. Therefore, fortifying effluent with mineral elements from weathered regolith offers potential for improvement of BLiSS streams.^3^ A limitation of the current study is that experimentation lasted only 24 h, whereas soil weathering typically occurs in geologic time scales; there is reason to believe that further weathering of minerals would continue given longer shaking times^44^ and that using previously established imaging methods for regolith may allow for understanding the shapes of the weathered regolith.^51^

## Conclusion

The hydrologic weathering of lunar and martian regolith simulants by BLiSS streams demonstrated increased plant essential nutrients and metals in solution due to highly soluble compounds and easily weatherable mineralogy. Multielement competitive sorption demonstrates low removal of P and K, but high supplementation of Ca, Mg, and S to the solution. The mechanistic differences between the organic BLiSS effluent and the inorganic nutrition solution were predominantly unaccounted for. It stands to reason that longer-term studies may follow up with the provided results to fully determine how supplementation of BLiSS solutions can be obtained from weathering of the regolith. Therefore, further experimentation that improves upon the extraction of elements of interest and increases the reaction time will be useful in fortifying the sustainability of human operations. The hydrologic weathering of sharp mineral edges also deserves quantitative investigation over time to understand whether reducing the abrasiveness of regolith offers an additional aspect of using BLiSS effluent on lunar and martian surface minerals.

## References

[1] Hoagland, D. R. ; Arnon, D. I. The water-culture method for growing plants without soil. Circular—California Agricultural Experiment Station; The College of Agriculture University of California, 1950; p 347.

[2] Ming, D. W. ; Henninger, D. L. Lunar base agriculture: Soils for plant growth. Lunar Base Agriculture: Soils for Plant Growth; Wiley, 1989; pp 1–255.

[3] Ming D. W., Henninger D. L. (1994). Use of
lunar regolith as a substrate for plant growth. Adv. Space Res..

[4] Anand M., Crawford I. A., Balat-Pichelin M., Abanades S., Van Westrenen W., Péraudeau G., Jaumann R., Seboldt W. (2012). A brief review of chemical
and mineralogical resources on the Moon and likely initial in situ
resource utilization (ISRU) applications. Planet.
Space Sci..

[5] Schwandt C., Hamilton J. A., Fray D. J., Crawford I. A. (2012). The production of
oxygen and metal from lunar regolith. Planet.
Space Sci..

[6] Rohde S., Wiltsche H., Cowley A., Gollas B. (2022). Dissolution and electrolysis
of lunar regolith in ionic liquids. Planet.
Space Sci..

[7] Meurisse A., Lomax B., Selmeci Á., Conti M., Lindner R., Makaya A., Symes M. D., Carpenter J. (2022). Lower temperature
electrochemical reduction of lunar regolith simulants in molten salts. Planet. Space Sci..

[8] Lomax B. A., Conti M., Khan N., Bennett N. S., Ganin A. Y., Symes M. D. (2020). Proving the viability
of an electrochemical process
for the simultaneous extraction of oxygen and production of metal
alloys from lunar regolith. Planet. Space Sci..

[9] Wilkerson R. P., Petkov M. P., Voecks G. E., Lynch C. S., Shulman H. S., Sundaramoorthy S., Choudhury A., Rickman D. L., Effinger M. R. (2023). Outgassing
behavior and heat treatment optimization of JSC-1A lunar regolith
simulant. Icarus.

[10] Verseux C., Poulet L., de Vera J. P. (2022). Bioregenerative
life-support systems
for crewed missions to the Moon and Mars. Front.
Astron. Sp. Sci..

[11] Espinosa-Ortiz E. J., Gerlach R., Peyton B. M., Roberson L., Yeh D. H. (2023). Biofilm
reactors for the treatment of used water in space:potential, challenges,
and future perspectives. Biofilm.

[12] Bullard, T. ; Smith, A. ; Hoque, B. ; Bair, R. ; Delgado-Navarro, M. ; Long, P. ; Uman, A. ; Yeh, D. ; Pickett, M. ; Roberson, L. A Prototype Early Planetary Organic Processor Assembly (OPA) Based on Dual-Stage Anaerobic Membrane Bioreactor (AnMBR) for Fecal and Food Waste Treatment and Resource Recovery. 50th International Conference on Environmental Systems, 2021; pp 12–15. https://hdl.handle.net/2346/87258 (accessed June 31, 2024).

[13] Smith, A. ; Bullard, T. ; Saetta, D. ; Hoque, B. ; Devito, C. ; Haarmann, K. ; Yeh, D. ; Bair, R. ; Long, P. ; Fehrenbach, M. ; Fischer, J. ; Roberson, L. Management of Fecal Waste Utilizing a Hybrid Organic Processor Assembly Unit Designed for Resource Recovery. 51st International Conference on Environmental Systems, 2022. https://hdl.handle.net/2346/89788 (accessed June 31, 2024).

[14] Saetta, D. ; Bullard, T. ; Smith, A. ; Yeh, D. H. ; Fischer, J. A. ; Koss, L. ; Monje, O. ; Finn, J. R. ; Roberson, L. B. Design and operation of Photomembrane Bioreactor (PMBR) to balance nitrogen in high-ammonia wastewater treatment effluents. 51st International Conference on Environmental Systems, 2022. https://hdl.handle.net/2346/89742 (accessed June 31, 2024).

[15] Saetta, D. ; Bullard, T. ; Smith, A. ; Yeh, D. H. ; Fischer, J. A. ; Dixit, A. ; Spern, C. ; Khodadad, C. L. ; Roberson, L. B. Survey of Microbial Community in Bioreactors Used for Bioregenerative Water Purification. 2023 International Conference on Environmental Systems, 2023. https://hdl.handle.net/2346/94700 (accessed June 31, 2024).

[16] Fischer, J. A. ; Koss, L. ; Monje, O. ; Finn, J. R. ; Saetta, D. ; Bullard, T. ; Smith, A. ; Yeh, D. H. ; Roberson, L. B. Lessons Learned from the Integration of Biological Systems in Series for Wastewater Treatment on Early Planetary Bases. 51st International Conference on Environmental Systems. 51st International Conference on Environmental Systems, 2022. https://hdl.handle.net/2346/89741 (accessed Aug 18, 2024).

[17] Hou Z., Gong Q., Liu N., Jiang B., Li J., Wu Y., Huang J., Gu W. (2023). Elemental Abundances of Moon Samples
Based on Statistical Distributions of Analytical Data. Appl. Sci..

[18] Morris, R. V. Surface exposure indices of lunar soils: A comparative FMR study. Lunar and Planetary Science Conference Proceedings, 1976; Vol. 1, pp 315–335.

[19] Chung D. H., Westphal W. B., Simmons G. (1970). Dielectric
properties of Apollo 11
lunar samples and their comparison with Earth materials. J. Geophys. Res..

[20] Strangway D. W., Chapman W. B., Olhoeft G. R., Carnes J. (1972). Electrical properties
of lunar soil dependence on frequency, temperature and moisture. Earth Planet. Sci. Lett..

[21] Loftus D. J., Rask J. C., McCrossin C. G., Tranfield E. M. (2010). The Chemical
Reactivity of Lunar Dust: From Toxicity to Astrobiology. Earth, Moon Planets.

[22] Gammage R. B., Holmes H. F., Fuller E. L., Glasson D. R. (1974). Pore structures
induced by water vapor adsorbed on nonporous lunar fines and ground
calcite. J. Colloid Interface Sci..

[23] Holmes H. F., Fuller E. L., Gammage R. B. (1973). Alteration
of an Apollo 12 sample
by adsorption of water vapor. Earth Planet.
Sci. Lett..

[24] Kaur J., Rickman D., Schoonen M. A. (2016). Reactive
Oxygen Species (ROS) generation
by lunar simulants. Acta Astronaut..

[25] Noble, S. The lunar regolith. In Lunar Regolith Simulant Workshop, 2009.

[26] Basu A. (2005). Nanophase
Fe0 in lunar soils. J. Earth Syst. Sci..

[27] Chevrier V. F., Fitting A. B., Rivera-Valentín E. G. (2022). Limited
Stability
of Multicomponent Brines on the Surface of Mars. Planet. Sci. J..

[28] Toner J. D., Catling D. C., Light B. (2014). The formation
of supercooled brines,
viscous liquids, and low-temperature perchlorate glasses in aqueous
solutions relevant to Mars. Icarus.

[29] Nair C. P. R., Unnikrishnan V. (2020). Stability of the Liquid Water Phase
on Mars: A Thermodynamic
Analysis Considering Martian Atmospheric Conditions and Perchlorate
Brine Solutions. ACS Omega.

[30] Chevrier V. F., Fitting A. B., Rivera-Valentín E. G. (2022). Limited
Stability
of Multicomponent Brines on the Surface of Mars. Planet. Sci. J..

[31] Jones E. G. (2018). Shallow
transient liquid water environments on present-day mars, and their
implications for life. Acta Astronaut..

[32] Adcock, C. T. ; Udry, A. ; Hauausrath, E. M. ; Tschauauner, O. Craters of the Moon National Monument basalts as unshocked compositional and weathering analogs for martian rocks and meteorites. Am. Mineral. 2018, 103 (4), 502–516. https://pubs.geoscienceworld.org/msa/ammin/article-abstract/103/4/502/529897/Craters-of-the-Moon-National-Monument-basalts-as?redirectedFrom=fulltext.

[33] Lasne, J. ; Noblet, A. ; Szopa, C. ; Navarro-González, R. ; Cabane, M. ; Poch, O. ; Stalport, F. ; François, P. ; Atreya, S. K. ; Coll, P. Oxidants at the Surface of Mars: A Review in Light of Recent Exploration Results.Astrobiology https://home.liebertpub.com/ast 2016, 16 (12), 977–996. DOI: 10.1089/AST.2016.1502. 27925795

[34] Kounaves S. P., Hecht M. H., Kapit J., Gospodinova K., Deflores L., Quinn R. C., Boynton W. V., Clark B. C., Catling D. C., Hredzak P., Ming D. W., Moore Q., Shusterman J., Stroble S., West S. J., Young S. M. M. (2010). Wet
Chemistry experiments on the 2007 Phoenix Mars Scout Lander mission:
Data analysis and results. J. Geophys. Res.
Planets.

[35] Pandey, A. ; Rampe, E. ; Morris, R. V. ; Peretyazhko, T. ; Niles, P. B. ; Archer, D. A. ; Casbeer, P. ; Clark, J. ; Sutter, B. ; Chipera, S. J. ; Vaniman, D. T. ; Pandey, A. ; Rampe, E. ; Morris, R. V. ; Peretyazhko, T. ; Niles, P. B. ; Archer, D. A. ; Casbeer, P. ; Clark, J. ; Sutter, B. ; Chipera, S. J. ; Vaniman, D. T. 55th Lunar and Planetary Science Conference, held 11-15 March, 2024 at The Woodlands, Texas/Virtual. LPI Contribution No. 3040, id.2301, 2024.

[36] Oze C., Beisel J., Dabsys E., Dall J., North G., Scott A., Lopez A. M., Holmes R., Fendorf S. (2021). Perchlorate
and agriculture on mars. Soil Syst..

[37] Stern J. C., Sutter B., Freissinet C., Navarro-González R., McKay C. P., Archer P. D., Buch A., Brunner A. E., Coll P., Eigenbrode J. L., Fairen A. G., Franz H. B., Glavin D. P., Kashyap S., McAdam A. C., Ming D. W., Steele A., Szopa C., Wray J. J., Martín-Torres F. J., Zorzano M. P., Conrad P. G., Mahaffy P. R., the MSL
Science Team (2015). Evidence for indigenous nitrogen in sedimentary and aeolian deposits
from the Curiosity rover investigations at Gale crater, Mars. Proc. Natl. Acad. Sci. U.S.A..

[38] Sutter B., McAdam A. C., Mahaffy P. R., Ming D. W., Edgett K. S., Rampe E. B., Eigenbrode J. L., Franz H. B., Freissinet C., Grotzinger J. P., Steele A., House C. H., Archer P. D., Malespin C. A., Navarro-González R., Stern J. C., Bell J. F., Calef F. J., Gellert R., Glavin D. P., Thompson L. M., Yen A. S. (2017). Evolved gas analyses of sedimentary
rocks and eolian sediment in Gale Crater, Mars: Results of the Curiosity
rover’s sample analysis at Mars instrument from Yellowknife
Bay to the Namib Dune. J. Geophys. Res. Planets.

[39] Sutter B., Archer P. D., Niles P. B., Ming D. W., Hamara D., Boynton W. V. (2024). Organic Carbon and
Ca-Rich Carbonate Detections in
Soils of the Northern Plains, Mars: Evaluation of Unreported Data
From the Mars Phoenix Scout’s Thermal Evolved Gas Analyzer
(TEGA). J. Geophys. Res. Planets.

[40] Zeng X., He C., Oravec H., Wilkinson A., Agui J., Asnani V. (2010). Geotechnical
Properties of JSC-1A Lunar Soil Simulant. J.
Aerosp. Eng..

[41] Ray C. S., Reis S. T., Sen S., O’Dell J. S. (2010). JSC-1A
lunar soil simulant: Characterization, glass formation, and selected
glass properties. J. Non. Cryst. Solids.

[42] Cannon K. M., Britt D. T., Smith T. M., Fritsche R. F., Batcheldor D. (2019). Mars global
simulant MGS-1: A Rocknest-based open standard for basaltic martian
regolith simulants. Icarus.

[43] Prieto A. L., Criddle C. S., Yeh D. H. (2019). Complex organic
particulate artificial
sewage (COPAS) as surrogate wastewater in anaerobic assays. Environ. Sci. Water Res. Technol..

[44] Eick M. J., Grossl P. R., Golden D. C., Sparks D. L., Ming D. W. (1996). Dissolution
of a lunar basalt simulant as affected by pH and organic anions. Geoderma.

[45] Marschner, P. Marschner’s Mineral Nutrition of Higher Plants, 4th ed.; Rengel, Z. , Ed.; Academic Press: London, 2022; Chapter 1, p 5.

[46] Judd, S. The MBR Book: Principles and Applications of Membrane Bioreactors for Water and Wastewater Treatment; Elsevier: Amsterdam, 2010.

[47] Jun D., Kim Y., Hafeznezami S., Yoo K., Hoek E. M. V., Kim J. (2017). Biologically
induced mineralization in anaerobic membrane bioreactors: Assessment
of membrane scaling mechanisms in a long-term pilot study. J. Membr. Sci..

[48] Le-Clech P., Chen V., Fane T. A. G. (2006). Fouling
in membrane bioreactors used
in wastewater treatment. J. Membr. Sci..

[49] Lyczko N., Nzihou A., Sharrok P. (2014). Calcium Phosphate
Sorbent for Environmental
Application. Procedia Eng..

[50] Davey H. P., Embery G., Cummins D. (1997). Interaction of zinc
with a synthetic
calcium phosphate mineral. Caries Res..

[51] Wilkerson R. P., Rickman D. L., McElderry J. R., Walker S. R., Cannon K. M. (2024). On the
measurement of shape: With applications to lunar regolith. Icarus.

